# Sphinganine-Analog Mycotoxins (SAMs): Chemical Structures, Bioactivities, and Genetic Controls

**DOI:** 10.3390/jof6040312

**Published:** 2020-11-24

**Authors:** Jia Chen, Zhimin Li, Yi Cheng, Chunsheng Gao, Litao Guo, Tuhong Wang, Jianping Xu

**Affiliations:** 1 Institute of Bast Fiber Crops and Center of Southern Economic Crops, Chinese Academy of Agricultural Sciences, Changsha 410205, China; chenjia01@caas.cn (J.C.); lizhimin@caas.cn (Z.L.); chengyi@caas.cn (Y.C.); gaochunsheng@caas.cn (C.G.); guolitao@caas.cn (L.G.); wangtuhong@caas.cn (T.W.); 2 Department of Biology, McMaster University, Hamilton, ON L8S 4K1, Canada

**Keywords:** sphinganine-analog mycotoxins, fumonisins, AAL-toxin, chemical structure, toxicity, genetics and evolution, biosynthesis

## Abstract

Sphinganine-analog mycotoxins (SAMs) including fumonisins and *A. alternata* f. sp. *Lycopersici* (AAL) toxins are a group of related mycotoxins produced by plant pathogenic fungi in the *Fusarium* genus and in *Alternaria alternata* f. sp. Lycopersici, respectively. SAMs have shown diverse cytotoxicity and phytotoxicity, causing adverse impacts on plants, animals, and humans, and are a destructive force to crop production worldwide. This review summarizes the structural diversity of SAMs and encapsulates the relationships between their structures and biological activities. The toxicity of SAMs on plants and animals is mainly attributed to their inhibitory activity against the ceramide biosynthesis enzyme, influencing the sphingolipid metabolism and causing programmed cell death. We also reviewed the detoxification methods against SAMs and how plants develop resistance to SAMs. Genetic and evolutionary analyses revealed that the *FUM* (fumonisins biosynthetic) gene cluster was responsible for fumonisin biosynthesis in *Fusarium* spp. Sequence comparisons among species within the genus *Fusarium* suggested that mutations and multiple horizontal gene transfers involving the *FUM* gene cluster were responsible for the interspecific difference in fumonisin synthesis. We finish by describing methods for monitoring and quantifying SAMs in food and agricultural products.

## 1. Introduction

Mycotoxins are secondary metabolites produced by various fungi. These metabolites have important ecological functions on living systems in their natural habitats. As secondary metabolites, mycotoxins are regarded as not essential for fungal growth or reproduction. However, their toxic effects to plants, animals, as well as humans are attracting increasing attention from chemists, biologists, food scientists, and healthcare professionals. Many fungi are capable of synthesizing mycotoxins, including certain saprophytic molds, poisonous mushrooms, human fungal pathogens, and plant fungal pathogens. Mycotoxins produced by plant pathogenic fungi can be divided into two groups: (i) host-selective (or host-specific) toxins (HSTs) and (ii) non-host-specific toxins (nHSTs), depending on whether they are specifically toxic to host plant (HSTs) or to a wide range of species (nHSTs). The known mycotoxins are typically low molecular-weight chemicals but with diverse structures and modes of actions. One group of mycotoxins are structurally analogous to sphingosine, the backbone precursor of sphingolipids that play essential structural and cellular roles in eukaryotic cells. These toxins are called sphinganine-analog mycotoxins (SAMs), with fumonisins and the *Alternaria alternata* f. sp. *Lycopersici* (AAL) toxins as the two most widely studied groups of SAMs. SAMs are toxic to plants and animals. They act by inhibiting the ceramide synthase (CerS), thereby influencing the sphingolipid metabolism and initiating apoptosis in animals and programmed cell death (PCD) in plants [1,2,3]. The objective of this paper is to provide an updated review on the structural diversity, syntheses, modes of action, and health impacts of SAMs.

The discovery of fumonisin was first reported in 1988 and the organism producing it was *Fusarium verticillioides* (syn. *Gibberella fujikuroi* mating population A, syn. *G. moniliformis* Wineland, syn. *F. moniliforme* Sheldon) [4]. Fumonisins have since been found to be produced by at least 18 species of the *Fusarium* genus, with *F. verticillioides* and *F. proliferatum* being the most prominent, and by three unrelated fungal genera, *Aspergillus* section *Nigri* (such as *Asp. niger*, *Asp. Welwitschiae* (syn. *Asp. awamori*) and so on, known as black aspergilli), *Tolypocladium* (*T. inflatum*, *T. cylindrosporum*, and *T. geodes*), and *Alternaria* (the tomato pathotype of *A. alternata,* formerly known as *A. arborescens*) [5,6,7,8,9,10]. Species of the *Fusarium* genus can be found as saprophytes in soil and as endophytes and pathogens of many plants worldwide. A common group of diseases caused by *Fusarium* pathogens is rotting that can happen to all tissues during all stages of plant development [11,12]. In addition, the *Fusarium* species can infect crops at the post-harvest period during storage [13]. The fungal propagules surviving in the soil can also infect new crop plants and can be carried to new fields by wind or by anthropogenic activities, such as when seedlings are transplanted [14]. *Fusarium* strains can synthesize fumonisins during all stages of their growth, including the saprophytic stage in the soil, during their pathogenesis, and as endophytes in different parts of plants, as well as during crop storage after harvest [15].

Fumonisins, as a nHST, are major contaminants of cereals and grains, including corn, rice, wheat, barley, rye, oat, millet, and products made based on these crops [16]. The consumption of food contaminated by fumonisins significantly increases health problems for humans, leading to a variety of cancers such as esophageal cancer and neurological defects [17,18]. For example, the International Agency for Research on Cancer (IARC) characterized fumonisin FB_1_ as a group 2B carcinogens for humans [16]. Fumonisins can also cause diseases and adverse effects in other species, especially in livestock when the feeds are contaminated [19]. Well-known diseases in livestock caused by fumonisins include leukoencephalomalacia in horses and pulmonary edema syndrome in pigs [20,21].

Similar to fuminisins, the AAL-toxins include a family of structurally analogous metabolites. AAL-toxins are a group of HST produced by the ascomycete fungal pathogen *A. alternata* f. sp. *Lycopersici*, the causal agent of tomato stem canker disease [22]. It should be noted that several other pathotypes of *A. alternata* could also produce other HSTs responsible for fungal pathogenesis on their specific host plants, respectively [23]. Unlike other HSTs produced by *A. alternata*, besides the susceptible tomato host, AAL-toxins can also affect many other weeds and crops of dicotyledonous species and at least 25 species of *Solanaceae* [24,25]. Furthermore, the tomato pathotype of *A. alternata* was also reported to produce fumonisins B (FBs) [8,26]. AAL-toxin and FBs were not only detected in the necrosis plant tissues and culture media inoculated by *A. alternata* but also in spores and mycelia of this pathogen [27]. However, AAL-toxin remains the only toxin as a pathogenicity factor for stem canker disease of sensitive tomato varieties, while fumonisins are toxigenic virulence factors [28].

Because of the adverse impacts of SAMs on animal and human health, these toxins are also attracting increasing attention from food inspectors and public health agencies. Over the last three decades, significant progress has been made in our understanding of SAMs. Our objectives of this review are to capture these developments on SAMs with regard to their chemical structural diversity, the relationship between structure and activity, PCD induction, detoxification, genetics and evolution of SAMs biosynthesis, and laboratory detections.

## 2. Chemical and Structural Properties

### 2.1. Chemical and Structural Properties of Sphingolipids

**Figure 1:**
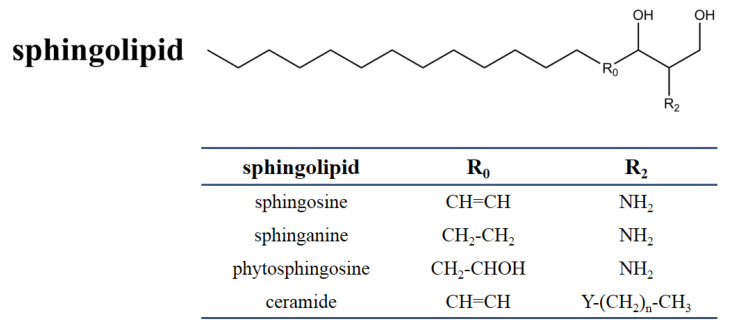
Chemical structure of sphingolipids. The table shows the different substituents in the chemical scaffold of the most essential sphingolipid.

SAMs have a distinct structural similarity to sphinganine (Figure 1). Sphinganine (dihydrosphingosine, DHS) is the simplest class of sphingolipids and has a backbone that consists of a linear aliphatic group with 18-carbon, an amino at C-2, and two hydroxyls (-OH) at C-1 and C-3, respectively. Phytosphingosine is obtained if a hydroxyl is introduced at C-4. Sphingosine consists of the sphinganine backbone but with a double bond at C-4. Ceramides are synthesized by linking an amide fatty acid at C-2 of sphingosine. Ceramides is a waxy lipid molecule, which is found in high concentrations in the membrane of eukaryotic cells. More complex sphingolipids can be formed by linking different chemical groups to hydroxyl (C1) of ceramides. Sphingolipids are one type of lipids widely found in their membranes in eukaryotes and a few prokaryotes, and they form complex and diverse interactions with other molecules [29]. Sphingolipids play important structural and functional roles, they are involved in a variety of signal transductions and crucial cellular processes [30,31]. For example, in humans, ceramides help form the skin’s barrier and regulate immune response, protecting the skin against environmental irritants, pollutants, and water loss. Without the proper ratio of ceramides on our epidermal cells, the barrier of the skin will be damaged, resulting in dryness, itching, and irritation [32].

### 2.2. Chemical and Structural Properties of Fumonisins

**Figure 2:**
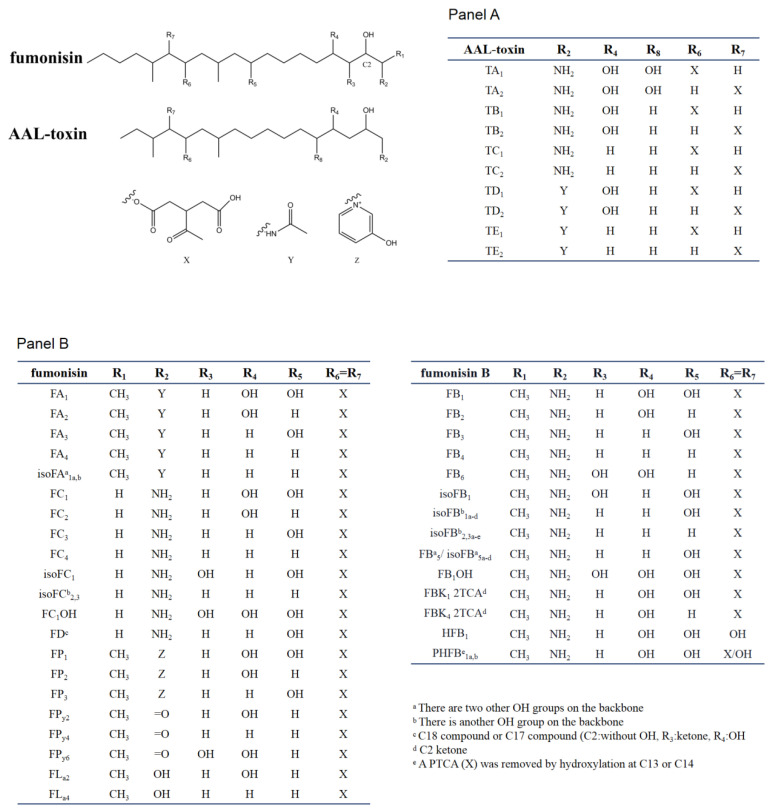
Chemical structure of sphinganine-analog mycotoxins (SAMs). Panel A shows the AAL-toxins, Panel B shows fumonisin. In the table of each panel, the different substituents present in the chemical scaffolds of individual compounds are shown.

SAMs consist of two main types of toxins, fuminisins and AAL-toxins. Fumonisins can be divided into seven groups (FA, FB, FC, FD, FP, FP_y_, and FL_a_). These groups differ in the nitrogen functional group and the length of the carbon backbone [5]. Most fumonisins contain a 19–20 (FD contain 17 or 18 carbon) linear backbone similar to sphinganine with one nitrogen functional group (except for FP_y_s and FL_a_s), two to four hydroxyl, two methyl, and two propane-1,2,3-tricarboxylic acid (PTCA) side chains esterified to the backbones [26,33]. The structural features of the seven groups of fumonisins are shown in Figure 2. Among them, the B group is the dominant one. For example, FB_1_ accounts for 70–80% of the total fumonisins produced by *F. verticillioides* and is the predominant toxic form [5]. FB_2_ and FB_3_ are isomers of each other but with one less hydroxyl group than FB_1_. The B series of fumonisins (FBs) are also the main food contaminants. Group A fumonisins (FA) are acetylated derivates of group B toxins, with lower toxicity and bioactivity than their FB counterparts [34]. Group C fumonisins (FC) have the same nitrogen functional group as FB_1_ but lack the terminal methyl group at C-1 [35]. Three forms of acetylated FC_1_ have been discovered in *F. oxysporum* [36]. Group P fumonisins (FP) have a nitrogen functional group of 3-hydroxypyridinium instead of the amino group in FB at the R_2_ position [37]. The FC and FP groups have similar phytotoxic and cytotoxic effects to those caused by FB_1_ or AAL-toxin [38]. Aside from these four main groups, there are several other lesser-known fumonisin analogs, with one or two PTCA replaced by a hydroxyl or carbonyl or other carboxylic acids group at C-13 and/or C-14 of the backbone (for example, HFB_1_, as show in Figure 2). Rheeder et al. summarized the 28 fumonisin analogs that have been characterized between 1988 and 2002 [5]. By reversed-phase high-performance liquid chromatography/electrospray ionization ion trap multistage mass spectrometry (RP-HPLC/ESI-IT-MS^n^), Bartok et al. detected 58 fumonisins (including FD) or fumonisin-like compounds from *F. verticillioides* in rice cultures, and 28 isomers of FB_1_ [33,39]. Indeed, the recent application of a semi-targeted method revealed over 100 structurally related compounds from SAMs-producing fungi, including a hydroxyl-FB_1_, and two new classes of non-aminated fumonisins (FP_y_s and FL_a_s) [26].

### 2.3. Chemical and Structural Properties of AAL-Toxin

The AAL-toxins have a structural similarity to fumonisins (Figure 2). The main difference between fumonisins and AAL-toxins is that AAL-toxins have one fewer PTCA side chain than fumonisins. The AAL-toxins have been divided into five pairs based on their side chain structures: A, B, C, D, and E pairs (TA, TB, TC, TD and TE). These pairs differ in their nitrogen functional group and hydroxylation at C-4 or C-5 positions of the backbone [40,41,42]. Each pair of AAL-toxins is composed of two regioisomers with PTCA esterified to C-13 or C-14 of the backbone, respectively. The TA pair is the major pair of toxins, with the TB and TC pairs formed by removing hydroxyl groups one by one from C-5 and C-4 of the TA pair. The TD and TE pairs were acetylated derivatives of TB and TC respectively, while the acetylated form of TA and keto derivatives of AAL-toxins (2-keto or 14-keto analogues predicted) were also found in 2015 [26]. These four regioisomeric pairs (TB, TC, TD, and TE) of AAL-toxins can all induce genotype-specific necrosis characteristics in tomato leaflets in the same pattern as that of the TA pair, but they differ as much as 1000-fold in their relative toxicity [42].

### 2.4. Chemical and Structural Properties of Analogs of SAMs

**Figure 3:**
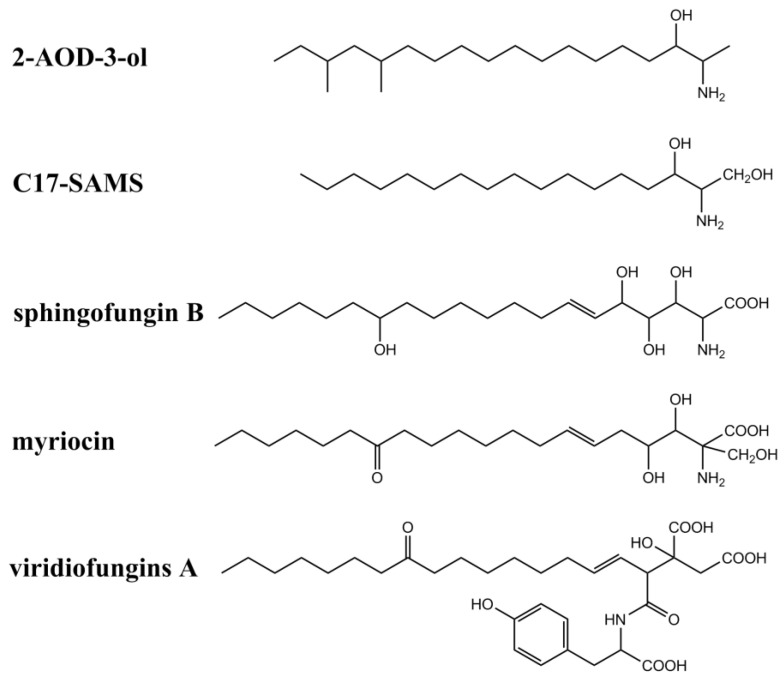
Chemical structures of other sphinganine-analog metabolites.

In addition to fumonisins and AAL-toxins, several fungal secondary metabolites have also been identified as structural analogs of sphinganine and CerS inhibitors (summarized in Figure 3 and Table 1). These metabolites include myriocins, sphingofungins, viridiofungins, 2-amino-14,16-dimethyl-octadecan-3-ol (2-AOD-3-ol), and a new C17-SAM identified from mussels contaminated by marine fungi including *Aspergillus*, *Fusarium,* and *Trichoderma.* Australifungin, a structurally unrelated mycotoxin produced by *Sporormiella australis,* was also shown to inhibit sphingolipid synthesis in plants, similar to those of SAMs.

Myriocins, sphingofungins, and viridiofungins inhibit serine palmitoyltransferase (SPT), while fumonisins, AAL-toxin, and australifungin inhibit sphinganine-N acyltransferase. Serine palmitoyl transferase, one of the key enzymes in the synthesis of sphingolipids, was also reported to play a positive role in PCD regulation. The increase of SPT activity promoted PCD in plants. In contrast, by inhibiting SPT activity, the excessive accumulation of sphingosine can be alleviated, leading to reduced PCD [43]. Therefore, myriocin are usually used as a SPT inhibitor to pretreat *Arabidopsis thaliana* and tomato plants to induce their resistance to FB_1_ and AAL-toxin, respectively [44,45].

## 3. Relationships between SAMs’ Structure and Biological Activities

The biological effects of SAMs, such as their toxicity, are similar among different SMAs. Many SAMs have a similar spectrum of susceptible plant species [34]. Tomato tissues and cells are similarly sensitive to AAL-toxins and to FB_1_ and FB_2_ toxins. In some other plants, AAL-toxins can cause necrotic cell death, similar to that of fumonisins [66]. For animal tissue cultures, the TA toxins can induce cytotoxicity in both rat liver and dog kidney cells as FB_1_ toxin [67,68]. Besides, AAL-toxin and *F. verticillioides* could also inhibit larval growth and reduced pupal weights of tobacco budworrn *Heliothis virescens* [69]. Such similarities have been attributed to the structural similarities between the SAMs and sphinganine. However, there are differences among SAMs in their biological effects and those differences are related to their structural differences. Below, we summarize the main findings in this area.

The amino functional group of SAMs is essential for their toxic activity. The peracetylated derivatives of AAL-toxins and FB_1_ are biologically inactive or have significantly reduced toxicity in both the plant bioassay and the animal tissue culture systems [66,70,71]. These results were consistent with initial reports on these toxins showing that blocking the free primary amines of AAL-toxins by specific reagents could abolish the biological activities of these toxins in plants [72]. In an in vitro test of rat primary hepatocytes, it was noted that the N-acetyl analogue of FB_1_, FA_1_, also showed CerS inhibition [68]. Later, FA was found to spontaneously undergo isomerization, rearranging its O-acetylation group to form different analogs. The impact of these rearrangement products on inhibition of CerS in rat liver slices also supported the important role of a primary amino for both CerS inhibition and toxicity [73]. Derivatization of the amino group with fluorogenic reagents also makes the FBs’ detection possible by the high-performance liquid chromatography (HPLC) assay [74]. FBs can bind covalently to proteins by reacting with amino groups in abiogenic conversions, which may increase the toxicity of those conversion products [75]. Similarly, the terminal amino group of FB_1_ can conjugate to bovine serum albumin (BSA) and work as an immunogen to produce monoclonal antibodies for enzyme-linked immunosorbent assay (ELISA) detection [76]. Amino group of fumonisins can also work as an electron donor and react with the electrophilic carbon within the isothiocyanate (ITC) group. Consequently, FBs can be degraded by fumigation treatment with ITC-containing compounds [77].

The hydrolysis product of FB_1_ (HFB_1_) was shown as less toxic than both FB_1_ and TA to plants [78]. Neither HFB_1_ nor the yeast sphingolipids (completely acetylated) contain PTCA. While both had adverse effects on duckweed growth, they showed lower phytotoxicity than TA and FB_1_ that contained one and two PTCA, respectively [79]. In contrast, the hydrolysis products of AAL-toxins largely maintain the toxicities of their parental compounds to the susceptible tomato lines [66]. These results indicate that PTCA is important to phytotoxicity of FBs and there is specificity of interaction between AAL-toxins and tomatoes.

Different from those in plants, an in vitro test using primary hepatocytes of rat showed that the HFBs had greater cytotoxicity than FBs. However, the HFBs could not initiate cancer development due to the lack of PTCA moiety, which was proposed to play an active role in the fumonisins absorption from the gut [70]. In the pregnant LM/Bc mouse model, HFB_1_ did not cause neural tube defects. In contrast, 10 mg of FB_1_/kg body weight of mice disrupted maternal sphingolipid metabolism, caused hepatic apoptosis in the female mice, increased fetus mortality, and reduced fetus weight [80]. In the SAMs-sensitive pig model, HFB_1_ was shown to have limited intestinal or hepatic toxicity but only slightly disrupted sphingolipids metabolism [81]. The toxic effects of FB_1_ and HFB_1_ exposure on intestinal barrier function and immunity in a pig intestinal porcine epithelial cells and porcine peripheral blood mononuclear cells co-culture model was also investigated. FB_1_ aggravated lipopolysaccharide (LPS)/deoxynivalenol (DON)-induced intestinal inflammation, while HFB_1_ showed less toxicity to the immune system [82]. In addition, when HFB_1_ and HFB_2_ were acylated by CerS, the N-acyl-metabolites were toxic in vitro to the human colonic cell line and in vivo to the intraperitoneal rat tissues [83].

Fumonisins are capable of binding to polysaccharides and proteins via their two PTCA side chains in thermal-treated food and form fumonisin artifacts [84]. The activities of SAMs vary depending on where hydroxylation occurs along the carbon backbone. For example, FB_2_ had a greater cytotoxic effect than FB_3_ and FB_1_ in primary rat hepatocytes [70]. However, different from most other side groups, the C-1 terminal methyl group, which differed between FC and AAL-toxin from other fumonisins, seemed not required for the biological activity in SAMs.

Similar symptoms but less phytotoxicities of SAMs were observed when long-chain sphingoid bases or simple sphingolipids were applied to duckweed, which indicated that the phytotoxicity of SAMs might be resulted from the accumulation of phytotoxic sphingolipid intermediates [71,85]. This result was consistent with the induction of PCD through ceramide-based signaling pathways (described below).

Although AAL-toxins and fumonisins are structurally related chemicals with similar phytotoxicity, the latter are 10 times less efficient. AAL-toxins have been considered to serve as an herbicide at a very low dosage against a wide variety of broadleaf weeds (e.g., jimsonweed, prickly sida, and black nightshade). However, monocotyledonous crops (e.g., maize, wheat, and resistant varieties of tomato) are tolerant to AAL-toxins [24,86,87]. Until 2013, the mode of action through CerS inhibition was not among the 21 molecular target sites of the commonly used herbicides. Using AAL-toxin as a lead compound has the potential to develop novel and safe bioherbicide, which has phytotoxicity but reduced or no mammalian toxicity [88,89].

## 4. Detoxification of SAMs

Using agricultural and manufacturing practices for preventing the spread and growth of toxin-producing fungi and limiting mycotoxin production is the preferred method to eliminate food contamination by fungal toxins at the pre-harvest period [90]. However, it is extremely difficult to completely prevent fungal pathogen growth and mycotoxin contamination in agricultural practices and in food storage and processing. Since the toxicity of SAMs is structurally dependent, our knowledge on the relationships between SAMs’ structure and biological activity provides clues for developing effective management strategies to minimize the impact of SAMs in food and feed products. Indeed, structural modifications such as hydrolysis have been demonstrated as effective at reducing the toxicity of SAMs [91].

Over the last three decades, chemical, biological, and physical strategies have been developed to degrade mycotoxins in food and feed products [92]. For example, nixtamalization was applied to reduce FBs by cooking fumonisin-contaminated maize with lime, as well as by using atmospheric ammoniation treatment [93,94,95]. Chlorine dioxide also showed the ability to degrade FB_1_ [96]. Two common cooking methods include extrusion and nixtamalization were shown to reduce the toxicity of FB_1_-contaminated corn [97]. Cold atmospheric pressure plasma was used as a physical treatment to successfully degrade pure FB_1_ and AAL-toxins within 60 s, while the presence of the matrix slowed down the degradation [98,99]. Ozone was applied to disrupt fungal cells of *Fusarium* and *Aspergillus* by oxidizing sulfhydryl and amino acid groups of enzymes or attacking the polyunsaturated fatty acids of the cell wall [100]. However, not all SAMs are susceptible to physical and chemical treatments. In addition, some of these treatments may also result in derivatives with unknown toxicity and be detrimental for the treated commodities, as shown in some cases [101,102].

Another method to reduce SAM toxicity is through microbial actions. Microorganisms can carry out biotransformation reactions to detoxify SAMs [91]. Such methods include deamination, acetylation, hydrolysis, glucosylation, and decarboxylation. For example, Benedetti et al. isolated a Gram-negative rod bacterial strain from soil capable of degrading fumonisin to four metabolites when fumonisin was supplied as the sole carbon source [103]. The bacterium *Sphingopyxis* sp. could detoxify fumonisin B1 by at least two enzymatic steps, including an initial de-esterification reaction followed by de-amination of hydrolyzed product [104]. Chlebicz and Śliżewska found that 12 strains of *Lactobacillus* sp. bacteria and 6 strains of *Saccharomyces cerevisiae* yeast could reduce the concentration of FB_1_ and FB_2_ by 40% [105]. Similarly, Burgess demonstrated that fumonisin-producing *Asp. welwitschiae* have the ability to produce enzymes to synthesize non-aminated fumonisins that are less toxic than FB, and that those enzymes could be used for fumonisin detoxification [106]. Indeed, using enzymes to detoxify by modification of chemical structures has become a promising method for mycotoxins control after grains harvest [107,108]. For example, fumD (carboxylesterase) from *Sphingopyxis* catalyze detoxification of FB_1_ to the hydrolyzed form by hydrolysis of both PTCA side chains. Then, the aminotransferase FumI could degrade FB_1_ by catalyzing the deamination of HFB_1_. FumD has also been tested for interference of fumonisins adsorption in turkey, swine, and pig [109,110,111]. Finally, several other enzymes such as manganese peroxidase from lignocellulose-degrading fungi and laccase from *Pleurotus eryngii* were all capable of degrading fumonisins [112,113].

Another potential method to reduce SAMs from food and feed products is to use adsorbent materials to soak up and remove the toxins. Many materials have shown the capacity to adsorb mycotoxins in vitro, thus the use of adsorbents in livestock diet as feed additives can potentially decrease the bioavailability of mycotoxins to humans and animals. As feed additives, cholestyramine, nanosilicate clay platelets, and refined calcium montmorillonite clay all reduced FB_1_ toxicosis [114,115,116]. Moreover, natural products such as the phenolic compound chlorophorin, honey, and cinnamon oil have all shown promise as fumonisin-reducing agents [117,118,119].

## 5. Programmed Cell Death and Sphingolipids

Almost all cells die eventually. There are four main types of cell death: necroptosis, pyroptosis, ferroptosis, and apoptosis, classified based on their distinct molecular and cellular processes and different outcomes. Apoptosis or programmed cell death (PCD) is a kind of cell suicide that strictly regulates cells that are no longer needed or are a threat to the wellbeing of multicellular organisms. Both plants and animals have PCD and they are functionally analogous to each other [120,121,122,123]. PCD plays essential roles to maintain normal physiological activities in multicellular organisms such as plants and animals and is an active self-regulating process to selectively eliminate redundant, aged, and damaged cells. PCD can be predicted for specific cells at defined developmental stages. However, PCD can also be induced by membrane-bound and cytosolic proteins stimulated by stress-induced signals. Such signals can trigger cell death via intricate cascades of transcriptional changes and post-translational protein modifications [122,124]. The characteristics of PCD include reduced cell volume, chromatin marginalization and condensation, nuclear lamina disassembly, DNA fragmentation, and apoptotic body formation, etc. [1].

PCD triggers and propagation involve many factors, including the expressions of certain cell surface receptors, transmembrane domains of several membrane proteins, intracellular proteins related to the propagation of death signals, secondary messengers including inositol triphosphate and ceramides, calcium (Ca^2+^) fluxes, reactive oxygen species (ROS), regulatory factors of cell cycle, and other suppressors or activators proteins. Many of these subcellular components, genes, and signal transduction pathways involved in PCD are functionally conserved across all domains of cellular organisms, from bacteria to fungi to plants and animals. However, there are differences in the actual mechanisms among organisms, as summarized in References [125,126].

Sphingolipids have been implicated to play an important role in cell growth, development, response to external environment, and PCD. As the main component of the cell membrane system, sphingolipids help to maintain the structural stability and transport of molecules across cell membranes [127,128]. In mammals, sphingolipids are especially abundant in the nervous system cells, with important functions in cell contact, growth, differentiation, communication, response to stress signals, and apoptosis [30,129]. In plants, sphingolipids are involved in response to both biotic and abiotic stresses, such as to pathogen infection, drought, and low temperature [31,130,131]. Indeed, the linkage of ceramide signaling to apoptosis has been widely reported in both plants and animals. Consequently, actions by SAMs to disrupt the functions of sphingolipids could have significant negative consequences. However, our knowledge about the roles of sphingolipids on apoptosis have also led to increasing interests on potential novel therapies using sphingolipids as treatment targets against degenerative and proliferative diseases in humans and animals, such as cancer and Parkinson’s disease [132,133].

A large number of studies have shown that sphingolipids could serve as critical secondary messengers in signal transduction to regulate PCD [134,135]. For example, in neutrophils, sphingolipids have been linked to increased superoxide formation and Ca^2+^ influx, which are universal signaling molecules involved in many cellular functions [136]. The induction of PCD in *Arabidopsis* by ceramides was also verified to be partly dependent on ROS in mitochondria or regulated by the release of Ca^2+^ [137,138]. In addition, increase of sphingosine level can activate the mitogen-activated protein kinase (MAPK) pathway in which MPK6 participates, promote the accumulation of sialic acid (SA), and then induce PCD [43]. Lachaud et al. found that the activated calcium-dependent kinase (CPK3) can regulate the process of sphingosine-induced PCD by dissociating CPK3 from the 14-3-3 protein-complex under increased calcium concentration induced by sphingosine. The activated CPK3 is then degraded followed by PCD induction [139].

Mutational studies of *Arabidopsis* and in vitro experiments have shown that ceramides, and free sphingoid bases such as sphingosine, sphinganine, and phytosphingosine, can all induce PCD. In contrast, phosphorylated products of these compounds can inhibit or alleviate PCD in plants [44,138,140,141,142]. However, phosphorylated sphingosine can inhibit the growth of yeast cells, which suggests that there are different mechanisms of action between plants and yeasts [143]. It is worth mentioning that not all ceramides can induce PCD. Nagano et al. found that when the C2 position of fatty acid in the side chain of ceramides was hydroxylated, PCD was inhibited rather than induced [144]. These results indicate that the occurrence of PCD in plants depends not only on the absolute content of sphingolipids, but also the relative ratios of various modified forms. In the next section, we will describe how SAMs are involved in PCD.

## 6. SAMs Trigger PCD through Ceramide-Based Signaling Pathways

In plants, pathogen invasions can lead to disruptions in host cellular homeostasis, and trigger cell death in susceptible varieties or even in resistant varieties with a hypersensitive response (HR). Because of its many similarities with PCD, HR is often considered as a form of PCD in plants. AAL-toxins, as the pathogenic factor of tomato stem canker disease, can induce PCD in sensitive tomato varieties resulting in fragmentation of chromosomal DNA and formation of apoptotic bodies in cells [145]. Similarly, when treated by FB_1_ produced by pathogenic *Fusarium*, *Arabidopsis* protoplasts showed symptoms similar to PCD in animal cells [146]. At the tissue and organ levels, *Arabidopsis* leaves treated with the FB_1_ toxin showed characteristic disease symptoms. Cells of the diseased leaves had overall phenotypes similar to HR, including callose accumulation, ROS production, and pathogenesis-related (PR) gene induction [147]. The damages caused by SAMs on host plants can further increase pathogen infection and colonization. Similar to those found in plants, SAMs can induce neuro-/renal-responses, heptatoxicosis, and neoplasms, as well as cell death in animals. The relationship between apoptosis and ceramide signaling has been established in both plants and animals in their response to SAMs [120]. For example, the induction of cell death in both tomato and African green monkey kidney (CV-1) cells occurred under similar toxin concentrations and time frames. For both types of cells, morphological markers characteristic of apoptosis were observed, including cells with positive terminal deoxynucleotidyl transferase end labeling (TUNEL), DNA fragmentations, and the formation of apoptotic-like bodies [145,148].

SAMs are structurally analogous to sphinganine and are thus effective inducers of PCD. The emerging mechanism of their actions is that SAMs can competitively bind to CerS in cells. Such binding leads to the accumulation of free sphingoid bases, the substrates of CerS, while ceramides as products of CerS were consumed and reduced, activating PCD in plant and animal cell lines [3,85]. For example, it was reported that FB_1_ is a potent competitive inhibitor of CerS from liver and brain microsomes in several mammalian cell lines [149,150]. An increase in sphinganine was observed in an in vivo test of CerS inhibition, as well as in FB-fed animals treated at high concentrations [2]. In addition, TA and FB_1_ can inhibit CerS in rat hepatocytes and green tomato fruits [150,151]. Furthermore, it was found that FB_1_ not only induced apoptosis in animal cells, but also altered cell morphology, cell–cell interactions, cell surface proteins behavior, protein kinase activity, and cell growth and viability in non-apoptotic cells [148,152]. In plant cells, after exposure to SAMs, sphingosine concentration increased significantly within a short time, followed by the accumulation of ROS in the cytoplasm and then apoptosis. These results suggested that the accumulation of sphingosine in cells was the upstream signal of ROS for cell death [43,44,85]. The induction of PCD by FB_1_ is also related to the accumulation of ceramides. In *A. thaliana,* there are two types of CerSs that use different substrates. Class I CerSs use sphinganine and C-16 fatty acyl-CoA as substrates, while class II use phytosphingosine and very long-chain fatty acyl-CoA as substrates. FB_1_ mainly inhibits the activity of class II CerSs. When treated with FB_1_, phytosphingosine in cells increases significantly. At the same time, as the product of a previous step, sphinganine also increases, which provides more substrates for class I CerS. Consequently, the products of class I CerS in cells increase, leading to induced PCD [153,154].

## 7. Plant Resistance to SAMs

Phytohormones are also involved in the defense reaction induced by SAMs. Changes in ethylene (ET) were first discovered in AAL-induced necrosis of tomato [155]. Alteration in ethylene perception in “never ripe” mutants of tomatoes can markedly alleviate the tissue damage caused by SAMs, which indicated an ethylene-associated signal transduction during plant cell death [156]. Later, a transcription analysis of AAL-toxin-induced cell death was carried out in *Arabidopsis*. Genes responsive to ROS and ET were among the earliest upregulated genes [157]. Mase used VIGS (virus-induced gene silencing) analyses and verified that the ET signaling pathway and MAPK cascades were required for AAL-toxin-induced PCD in tobacco [158]. By SA-mediated ET suppression, glutathione (GSH) may be involved in resistance primarily against AAL-toxin-induced stress in Arabidopsis [159].

Unlike ethylene in host basal defense responses against the tomato pathotype of *A. alternata*, the jasmonate (JA)-dependent signaling pathway is not involved in host defense against the toxigenic *A. alternata* pathogen. JA affects pathogen acceptability via a toxin-independent mannerin in the interactions between plants and toxigenic necrotrophic fungal pathogens. It may act upstream of ethylene biosynthesis in AAL-toxin-triggered tomato cell death [160,161]. Later, a comparative proteomics analysis revealed that the COI1 (coronatine insensitive 1, JA receptor)-dependent JA pathway enhances AAL-toxin-induced PCD of tomato through regulating the redox status of the leaves, other phytohormone pathways, and/or important PCD components [162].

The sensitivity of tomato plants to the fungal pathogen *A. alternata* f. sp. lycopersici is controlled by the *Alternaria* stem canker resistance locus (*Asc*-locus) on chromosome 3 [163]. Mutations of tomato *Asc* locus gives resistance to the pathogen, while overexpression of the tomato *Asc-1* gene mediates high insensitivity to SAMs in tomato and confers resistance to pathogen infection in sensitive *Nicotiana* plants [164,165]. *Asc-1* is a homolog of the yeast longevity assurance gene *LAG1*, which encodes components of sphinganine N-acyltransferase. This resistance gene could prevent the disruption of sphingolipid metabolism during AAL-toxin-induced PCD. Both *Nicotiana* and *Lycopersicon* genera belong to Solanaceae. In tomato, insensitivity to SAMs and susceptibility to the pathogen is determined by *Asc-1* [166]. In contrast, the SAM-sensitive species in the *Nicotiana* (except for *N. umbratica*) still have *Asc-1* homologs and are resistant to *A. alternata* f. sp. lycopersici infection with HR, which indicates an additional (non-host) resistance mechanism between *Nicotiana* and this pathogen [167]. The multilayered defense systems also exist in *Arabidopsis* non-host resistance to *A. alternata* [168]. Similarly, although many *Fusarium* species produce fumonisin, they cannot infect AAL-sensitive tomato. This non-host resistance includes a multi-layer defense system involving both pre- and post-invasion, and help plants defend against various pathogens [169]. In addition, Zélicourt demonstrated that two of three Lag1 homologs in the *Orobanche cumana* genome were responsible for an enhanced sensitivity to AAL-toxin [170].

Aside from the above-mentioned genes, several other genes were identified from Arabidopsis and found to be involved in the AAL-induced PCD pathway, including Zinc *A. thaliana* 11 (a zinc finger protein ZAT11), fbr41 (FB_1_ Resistant41), and baculovirus p35 gene (inhibitor of a class of cysteine proteases). All of them showed protective effects on AAL-toxin-induced cell death and pathogen infection in plants [171,172,173]. Discovery of resistant genes has provided a potential strategy for SAMs’ control in crop production by plant transgenic modification.

Because of the high toxicity of fuminisins, especially FB_1_, a large number of studies have focused on them. So far, the mechanism of FB_1_ toxicity has been centered around its structural resemblance with sphinganine and consequent competitive inhibition of CerS and the disruption of lipidomic profiles. However, there is emerging evidence suggesting that FB_1_ can disrupt mitochondrial function and generate excessive toxic ROS.

Table 2 shows a list of reviews summarizing the latest advances related to fumonisins, in their assessment, biosynthesis, detection, crop breeding of resistant varieties, and toxicity.

## 8. Genes Responsible for SAMs Production

In fungi, genes directly involved in the biosynthesis pathway of the same secondary metabolite are usually located at adjacent positions in gene clusters in the genome [191]. These genes are often co-expressed and co-regulated. The genes involved in fumonisin biosynthesis fit this general pattern. Specifically, *Fusarium* species capable of producing fumonisins typically contain one gene cluster involved in their synthesis, called the *FUM* gene cluster. At present, a total of 21 genes have been identified in *FUM* gene clusters of various species and verified to be involved in fumonisin biosynthesis and regulation or self-protection using a variety of approaches, such as gene knockouts, domain swapping, and heterologous expression (Table 3) [192,193,194,195,196,197]. In *F. verticillioides*, the *FUM* cluster responsible for fumonisin B biosynthesis includes 17 genes [193,197,198].

**Figure 4:**
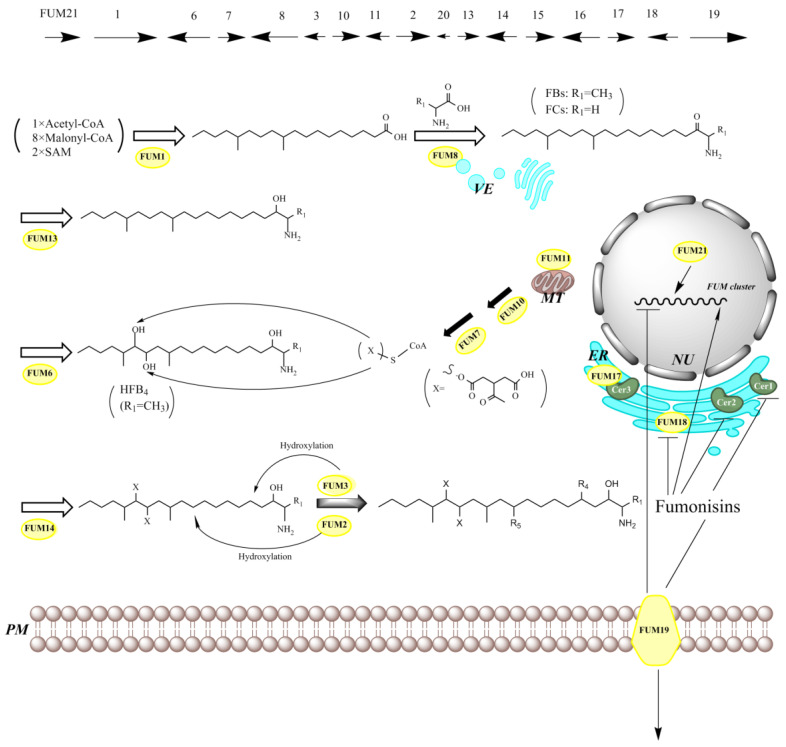
The *FUM* gene cluster, the proposed pathway of fumonisin biosynthesis, and the proposed mechanism for self-protection against fumonisins toxicity by the toxin-producing fungi (FBs and FCs). SAM, S-adenosyl methionine; VE, vesicles; MT, mitochondrion; ER, endoplasmic reticulum; NU, nucleus; PM, plasma membrane.

As described previously, SAMs are polyketide-derived compounds with structural similarity to sphinganine. Polyketides are synthesized by polyketide synthases (PKSs), which are large multifunctional enzymes. *FUM1* is a PKS gene previously designated as *FUM5* [199]. PKS encoded by *FUM1* catalyzes the synthesis of an octadecanoic acid precursor as the initial step for FB biosynthesis in *Fusarium* spp. [200,201]. The proposed biosynthetic pathway of FB is described in Figure 4 [197]. The precursor mentioned above undergoes condensation with L-alanine to synthesize the polyketide backbone, this reaction was catalyzed by the aminotransferase Fum8 [192,201]. In *F. oxysporum* strain O-1890, the orthologue of *FUM8* determines that *Fusarium* produces predominantly FCs [195]. The *fum8* deletion in some stains of *A. welwitschiae* is also considered to be associated with the loss of FB_2_ production [202].

A likely mitochondrial carrier protein encoded by *FUM11* transport the substrate tricarboxylate for Fum7 (dehydrogenase) and Fum10 (acyl-CoA synthase) to produce CoA-activated tricarballylic acid, which are attached to the polyketide backbone by Fum14 (condensation-domain protein) [192,194]. The ensuring steps of fumonisin biosynthesis involving various modifications of the backbone (including primarily hydroxylation) were catalyzed by several enzymes, such as Fum6/Fum12/Fum15 (cytochrome P450 monooxygenase), Fum2 (hydroxylase of C10), Fum13 (short-chain dehydrogenase/3-ketoreduction), and finally, Fum3 (hydroxylase of C5, dioxygenase), catalyzed by *FUM9*-encoded protein (alleles of *FUM3*) [192,193,203,204,205,206].

In addition, *FUM21* encoding a GAL4-like Zn(II)2Cys6 transcription factor was verified to be involved in the regulation of fumonisin synthesis. However, it seemed that the deletion of *FUM16* had no apparent effect on fumonisin production in *F. verticillioides* [194,198,207,208,209]. Recently, *FUM17*–*FUM19* in *F. verticillioides* were found to help the fungus to avoid its own toxicity during fumonisin production. Fum19 is an ATP-binding cassette transporter (ABC transporter) and acts as a repressor of the *FUM* gene cluster. *FUM17* and *FUM18* are CerS homologs. *FUM18* could fully complement the yeast CerS null mutant *LAG1*/*LAC1*, while co-expression of *FUM17* and CER3 partially complemented. Both the Fum17 and Fum18 proteins enable *F. verticillioides* to increase its resistance of fumonisin by providing *FUM* cluster-encoded CerS activity as a first level of self-protection [197].

Aside from the *FUM* genes, other genes like *FST1* (transporter), *FUG1* (transcription or signal transduction factors), *CPP1* (protein phosphatase type 2A catalytic subunit), and *FvVEl* (regulator) in *F. verticillioides*, *PKS3* and *PKS11* in *F. proliferatum*, and GATA-type transcription factors *AreA* and *AreB* (known as the global nitrogen regulators) in *F. fujikuroi* have also been demonstrated to have an important role in fumonisin biosynthesis and regulation [209,210,211,212,213,214]. In addition, a degenerated, over-represented motif which is potentially involved in the cis-regulation of *FUM* genes and fumonisin biosynthesis was also identified from both *F. verticillioides* and *Asp. niger*, while it was not found in fumonisins non-producing fungi containing various *FUM* homologues [215].

Several abiotic and biotic factors have been found to affect the expression of *FUM* genes and regulate biosynthesis of fumonisin. These factors include water activity, temperature, carbon sources and other nutrients, host plant species and varieties or their extracts, and plant age [216,217,218,219,220]. Mature plants and extracts from those plants are often associated with higher concentrations of SAMs. It has been suggested that harvesting the crop at earlier stages other than full maturity could be one of the strategies to control fumonisin contamination [221,222].

The genome sequencing and analysis of *Asp. niger* revealed that its genome contained a gene cluster (*fum* cluster) homologous to the *FUM* cluster in *Fusarium* species (shown in Table 3). Specifically, 12 homologues of the fumonisin synthesis genes were found, including *fum1, fum3, fum6, fum7, fum8, fum10, fum13* to *fum16, fum19,* and *fum21* genes [208,223,224]. This gene cluster is also found in fumonisin-producing isolates of *Asp. welwitschiae* but is absent from the genomes of other sequenced *Aspergilli* that do not produce fumonisin, such as *Asp. fumigatus*, *Asp. oryzae*, and *Asp. nidulans* [7,208]. In addition, homologs of multiple *fum* genes have been found in several other *Aspergilli* spp. but where no fumonisin production has been detected (summarized in Table 4). Some of the *Aspergullus* spp. contain genes that are unique to them. For example, a dehydrogenase gene (*sdr1*) of a short-chain length was found in the *fum* cluster of *Asp. niger* but is absent in the *FUM* gene cluster of *Fusarium* spp. In contrast, the *Fusarium FUM2* gene with a function of hydroxylation at the C-10 backbone position of fumonisin is absent in the *Asp. niger fum* cluster [207,208]. This result is consistent with the study that shows that *Asp. niger* only produces fumonisins FB_2_, FB_4_, and FB_6_, which lack a hydroxyl at C-10 [7,225,226]. However, isolates of several black aspergilli (including *A. niger*, *Asp. foetidus,* and *A. tubingensis*) isolated from peanuts and maize also produced FB_1_ and FB_3_, consistent with a complex biosynthesis pattern of the fumonisins in *Aspergilli* spp. [227].

**Table 3:** Homologous genes and their functional roles in the biosynthesis of SAMs.

Homologue of Cluster Genes			Predict Gene Product and Function	Reference
fumonisin		AAL-toxin		
*Fusarium* spp.	*Aspergillus* spp.	*A. alternata*		[169,224]
*FUM1* (fumonisin biosynthetic gene 1, previously designated as *FUM5*))	*fum1*	*ALT1* (AAL-toxin biosynthetic gene 1)	polyketide synthase	[12,199,200]
*FUM2*	absent		Dioxygenase for hydroxylation of C10	[228]
*FUM3*	*fum3*		Dioxygenase for hydroxylation of C5 (the same gene as *FUM9*)	[205,228,229]
*FUM4*			Not clear	[230]
*FUM6*	*fum6*	*ALT2*	Cytochrome P450 monooxygenase–reductase fusion proteins for hydroxylation of C14/C15	[192,231]
*FUM7*	*fum7*	*ALT3*	Type III alcohol dehydrogenases for PTCA (propane-1,2,3-tricarboxylic acid) side chain formation	[192,194,232]
*FUM8*	*fum8*		α-oxoamine synthase and homologous for amino transfer and FBs/FCs production	[192,195]
*FUM10*	*fum10*		Fatty acyl-CoA synthase for PTCA esterification	[193,194]
*FUM11*			mitochondrial transport protein for PTCA transport	[193,194]
*FUM12*			cytochrome P450 monooxygenases	[193]
*FUM13*	*fum13*	*ALT6*	Short-chain dehydrogenase/ketoreductase of C3	[193,204,206]
*FUM14*	*fum14*		Non-ribosomal peptide synthetase for PTCA esterification	[193,194,233]
*FUM15*	*fum15*		Cytochrome P450 monooxygenases	[193]
*FUM16*	*fum16*		Fatty acyl-CoA synthetase	[193,194,224]
*FUM17*			CerS for self-protection against fumonisins	[193,197]
*FUM18*			CerS for self-protection against fumonisins	[193,197]
*FUM19*	*fum19*		ABC transport protein as a repressor of FUM gene cluster	[193,197]
*FUM20*			Not clear	[234]
*FUM21*	*fum21*	*ALT13*	Zn(II)2Cys6 transcription factor	[198]
absent	*SDR1*		Short-chain dehydrogenase/reductase (SDR)	[7]

AAL-toxins are produced by *A. alternata* f. sp. *Lycopersici,* a specific pathotype of a common plant fungal pathogen in a genus different from *Aspergillus* and *Fusarium.* This pathotype can produce polyketide-derived compounds similar in structure to fumonisins produced by *Fusarium* species. The *ALT* (AAL-toxins synthesis) genes are also located as a cluster on a conditional disposable (CD) chromosome of ~1.0 Mb in all strains of the tomato pathotype of *A. alternata* from different countries [237]. Such CD chromosomes carrying a toxin biosynthesis gene cluster were also found in other pathotypes of *A. alternata* [169]. They control other HSTs production and pathogenicity to their host. They could maintain stably in a new genetic background to an expanded range of pathogenicity, which was verified by a protoplast fusion test [238]. The AAL-toxin gene cluster includes at least 13 genes in a 120 kb region, some of which showed significant similarity to the *FUM* gene cluster consisting of 17 genes in a 45.5 kb region. However, the arrangement of genes in the *ALT* and *FUM* clusters differs between these two groups of fungi. In addition, in one strain, As-27 of *A. alternata*, there were two sets of the AAL-toxin biosynthetic gene cluster on the CD chromosome [169]. The synthesis of AAL-toxins was found to be initiated by *ALT1*-encoded PKSs to produce the aminopentol backbone, which was then modified by other enzymes [239]. The functional similarity between *ALT1* and *FUM1* was confirmed when fumonisin biosynthesis in *FUM1*-disrupted *F. verticillioides* was restored when complemented by the *ALT1* from *A. alternata* [240]. Similarly, expression of *ALT1* and production of AAL-toxins were also found to be regulated by the global regulator *LaeA* [241]. AAL-toxin accumulation also benefits from high water activity (0.995 aw) and high temperature (above 30 °C) during the incubation period of the pathogen [242].

Interestingly, a PKS gene similar to *FUM1* and orthologs of the *FUM* gene cluster were found in the genome of *Cochliobolus* spp. by phylogenetic analysis of fungal polyketide. These fungi were also speculated to produce a fumonisin or other SAMs [243,244]. To predict the potential distribution of SAM production in fungi, Kim et al. proposed a hypothesis on SAM biosynthetic gene clusters based on fumonisin biosynthesis model. This putative gene cluster should include a PKS, an aminotransferase, and a dehydrogenase gene. Their model showed that sixty-nine species of the *Fusarium* genus and species of twenty-four other fungal genera were predicted to have at least one SAM cluster [245].

## 9. Evolution of SAMs Production

Horizontal gene transfer (HGT) has been proposed as a major mechanism responsible for the acquisition and evolution of fumonisins and AAL-toxins biosynthetic gene clusters among divergent fungi [7,9,169,224]. In *Fusarium*, genome sequence analyses revealed that the fumonisin biosynthetic genes (*FUM*) are clustered and show a consistent gene organization among most species. For example, the *FUM* clusters in *F. oxysporum*, *F. proliferatum,* and *F. verticillioides* exhibit relatively little variability, with the order and orientation of genes within the clusters all being the same as each other. In addition, their sequence variability among the orthologues of coding regions from *F. oxysporum* and *F. verticillioides* is relatively low [193,195]. The two different species of *Aspergillus* that produce fumonisins, *Asp. niger* and *Asp. welwitschiae*, are also similar to each other in their gene order but different from that of the *FUM* cluster in *Fusarium* [224]. At present, the tomato pathotype of *A. alternata* was the only species of genus *Alternaria* capable of producing SAMs and this pathotype has clustered genes (involved in AAL-toxin biosynthesis) similar to the *FUM* cluster in *Fusarium* [169,237]. Together, the gene structure and sequence analyses suggested that the SAMs biosynthetic gene cluster likely originated in *Fusarium* and transferred to *Asp. niger* and *A. alternata* by HGT. The similarities in chemical structure and cytotoxicity on plants and animals between fumonisins and AAL-toxins are also supportive of this hypothesis. However, the differences between *FUM* clusters and the AAL-toxins biosynthetic gene cluster also suggested that there has been significant divergence between them.

Analyses of the *FUM* gene cluster among *Fusarium* species also revealed evidence for gene gain, loss, and mutations of different genes. For example, not all *Fusarium* species can produce fumonisins. Even for species that can synthesize fumonisins, some strains produce more than others under the same experimental conditions, while other strains do not produce the toxins at all [9,196]. Indeed, for certain strains, while the *FUM* genes were detected, there was no detectable fumonisin. This was likely due to the accumulation of mutations leading to the *FUM* genes being nonfunctional. For example, several mutations have been found in *FUM7* and *FUM21* in *F. fujikuroi* [246]. Furthermore, several fumonisin-non-producing *Fusarium* species lack the fumonisin biosynthetic genes but retain homologs of several genes that flank the Fum cluster in *F. verticillioides* [247]. Interestingly, the flanking regions of the *FUM* cluster often differ between species, consistent with the independent origins of the *FUM* cluster, including independent acquisition and/or loss of the gene cluster by fumonisin-producing species [195]. For example, fumonisin-non-producing strains of *F. verticillioides* isolated from banana did not contain the functional fumonisin biosynthetic gene (*FUM*) cluster but did contain portions of *FUM21* and *FUM19* flanking the cluster, both of which are the terminal genes at each end of the *FUM* cluster. However, the banana strains are still pathogenic to banana, but they do not show the same pathology as the fumonisin-producing strains do on maize. When a banana strain was co-transformed with two overlapping cosmids containing the entire *FUM* gene cluster, fumonisin production and pathogenicity on maize seedlings were recovered [248]. Similar to *Fusarium*, FB-non-producing isolates of *Asp. niger* or other *Aspergillus* species also had an intact *fum* cluster or multiple patterns of *fum* gene deletion, respectively (shown in Table 4). Similarly, the AAL-toxin-production gene cluster in *A. alternata* was likely derived from *Fusarium* species by an HGT event. Evidence from the *ALT* gene cluster distributed in isolates of *A. alternata* also supports this hypothesis of HGTs within AAL-toxin-producing pathogens [237]. Together, these results suggest that there have been multiple HGTs of the cluster between species, as well as duplication and loss of the whole or part of the cluster after acquisition [9].

The hypothesis that multiple HGTs were involved in generating the current distribution of *FUM* gene clusters is further supported by phylogenetic studies. Specifically, phylogenetic trees based on genes from the *FUM* cluster often do not parallel that of the *Fusarium* species tree based on other genes [9]. In *Fusarium*, the translation elongation factor (tef-1α) gene is the most commonly used marker gene for taxonomic studies. However, sequences of the tef-1α are often insufficient for distinguishing fumonisin-producing isolates from different countries and/or host plants. In contrast, DNA sequence polymorphisms based on *FUM1* often provide better resolutions among pathotypes [196,249,250,251,252,253]. For example, phylogenetic analysis of 38 *F. proliferatum* isolates originating from different hosts showed that sequence variation among strains in the *FUM1* gene was correlated with that of the host plants. Specifically, phylogenetic analysis based on the partial *FUM1* sequences differentiated the host-related groups more clearly than that based on tef-1α sequences. The best distinguished group consists of garlic-derived isolates and formed a separate branch on a *FUM1*-based dendrogram [196,254]. Similarly, *FUM1* sequence divergence analysis on *F. proliferatum* and *F. verticillioides* strains isolated from pea also formed a distinct group when compared to strains derived from different host species [253].

Aside from gene differences among isolates from different host plants, variations of both toxigenic potential and growth patterns may also differ between isolates derived from the same host plants. While no difference was observed in FB levels measured among pea seeds, the FB productions differed between selected strains of *F. proliferatum* in rice cultures [253]. Among all these test isolates of *F. proliferatum*, the most varied group of isolates found so far were those isolated from maize [196,254]. Both the inter- and intra-specific variation in FBs synthesis level can at least partly be explained by the sequence differences inside the FUM cluster.

In summary, the analyses so far suggested that the *FUM* gene cluster was responsible for fumonisin biosynthesis. Mutation and deletion of some or all of the genes in the cluster could result in limited or no production of fumonisin, leading to a weaker disease development of the pathogen on the host plant. Sequence analyses showed that *A. alternata* has likely gained the ability for AAL-toxin production due to HGT of the SAMs gene cluster from fumonisin-producing *Fusarium* species followed by independent evolution in pathogen–host interaction. The divergent patterns of toxin biosynthesis gene sequence divergence may explain the differences between fumonisins and AAL-toxins in both their productions and their impacts on host–pathogen interactions.

## 10. Detection Method of SAMs

A variety of methods have been developed to detect SAMs, including HPLC with fluorescence/evaporative light scattering detection or mass spectrometry (MS), thin-layer chromatography (TLC), ELISA, Fourier transform near infrared (FT-NIR) spectroscopy, and so on [255,256,257,258,259,260,261,262,263]. While these traditional analytical methods were designed to detect and quantify known compounds for which standards are available, there is clear evidence that many unknown derivatives may exist in food and food products and some of these could be toxic to animals, including humans [39]. In 2015, a semi-targeted method combining product ion filtering and rapid polarity switching was designed for fast detection of all known fumonisins and AAL-toxins. Some new structurally related emerging toxins were also discovered by this method [26].

Aside from method development that targets potentially novel SAMs not reported before, there are also developments for efficient methods that target the detection of known SAMs. Indeed, several new methods based on immunoassay were developed for simple, rapid, and ultrasensitive on-site quantification of SAMs. For example, one method used chemiluminescent biosensors integrating a competitive lateral flow immunoassay and a charge-coupled device camera to detect FBs [264,265]. Another method uses gold nanoparticles or quantum dots nanobeads based on monoclonal antibodies against fumonisin and allows rapid detection of this mycotoxin in one step [266,267,268]. Furthermore, a loop-mediated isothermal amplification (LAMP) assay, based on the detection of *fum10*, could specifically detect the genes involved in FB_2_ biosynthesis in *Aspergillus* species that could be evaluated using the naked eye in a short time [269]. This method was also applied to detect FB_1_ targeting the *FUM1* gene in *Fusarium* [270]. The direct detection of genes involved in the biosynthesis of SAMs in agriculture production systems allows broad evaluations of the potential fumonisin-producing strains in food and feed products. To this end, multiplex PCR has shown great promise for detection of multiple fumonisin-producing *Fusarium* and *Aspergillus* species [271,272,273].

While the current focuses are on the known SAMs with known toxicities, there is increasing evidence that some of these toxins are masked and not easily detected or quantified. To ensure food safety, both the free forms and the masked forms of mycotoxins should be detected and quantified. The masked mycotoxins are usually modified forms of the mycotoxins by plant enzymes during infection and are not typically detectable during routine analysis. For example, the masked mycotoxins may conjugate with polar substances, store in the vacuole in the soluble form, or bind to macromolecules, and thus change their physiological properties. While the masked mycotoxins are often less toxic than the unmasked forms, they could be easily converted to the unmasked toxin forms, including during food digestion [274,275]. The most representative masked mycotoxins are the modified forms of Zearalenone (ZEN), DON, and fumonisins [276,277,278,279]. The so-called hidden fumonisins could form non-covalent bonds with food macro-constituents such as those in starch-based products. In certain situations, the masked fumonisins may be present in food at quantities much higher than the free forms. Many factors could influence the relative portions of the SAMs in masked forms, including crop growth conditions and food storage and processing techniques [279,280,281,282]. These hidden dangers require that novel method(s) be developed to allow the detection and quantification of the masked forms of SAMs as well as other mycotoxins.

## 11. Concluding Remarks

SAMs are highly toxic fungal compounds that have attracted significant attention from broad communities. They have toxicities to both plants and animals. These SAMs-producing pathogens are widely distributed in nature and closely related to agricultural production. Since its discovery in the mid-1980s, fumonisin has been among the mycotoxins with the greatest concern. As of now, Scopus database citations of fumonisin are above 5000, including 500+ reviews. SAMs are a series of compounds with structural similarity to sphingosine. As detection methods improve, additional new analogs have been continuously discovered. Research so far has shown that the toxicity and activity of SAMs are dependent on their structures. In this review, we summarized the detoxification method based on their structural properties using chemical, biological, and physical strategies. The toxicity of SAMs is mostly due to their inhibitory effects on CerS, disruption on sphingolipid metabolism, and initiation on PCD. Except for the adverse effect of SAMs on animals and humans, its phytotoxicity (e.g., AAL-toxin) could potentially be used for herbicide development and a model for studying the molecular mechanism of PCD in plants. Horizontal gene transfers on the SAMs biosynthesis gene cluster seemed widespread in these toxin-producing fungi, especially among *Fusarium* spp. Such phylogenetic distribution patterns suggest that there are potentially other fungi capable of producing SAMs, including their various modified forms. These and other issues require continued efforts from the scientific community on SAMs.

## Figures and Tables

**Table 1:** Analogs of sphinganine-analog mycotoxins (SAMs), their fungal producer(s), and their activities.

Analog of SAMs		Fungi/Origin	Activities	Scopus Citation (Review)	Reference
Myriocins (thermozymocidin, ISP-I)		*Myriococcum albomyces* *Melanconis flavovirens* *Isaria sinclairii*	Antifungal activity Inhibitor of serine palmitoyltransferase (SPT) Immunosuppressive activity Protective effect on hepatotoxicity Relieve fumonisin B_1_ (FB_1_)-induced toxicity and cell death Multi-pharmacological function on human	421(34)	[46,47,48,49,50,51,52,53]
Sphingofungins	E/F	*Paecilomyces variotii*	Inhibitor of SPT Antifungal activity	65(15)	[54,55,56,57,58]
	A/B/C/D/I	*Asp. fumigatus*			
	G/H	*Asp. penicilliodes*			
Viridiofungins	A/B/C	*Trichoderma viride Pers*	Inhibitors of SPT and squalene synthase Antifungal but lack antibacterial activity	21(5)	[59,60,61]
		*Tri. harzianum*			
Australifungin		*Sporormiella australis*	Inhibitors of sphinganine-N acyl transferase Antifungal activity, phytotoxicity	26(7)	[62,63]
2-AOD-3-ol		*F. avenaceum*	Animal cell toxicity as fumonisin B	5	[64]
C17-sphinganine analog mycotoxin		Contaminated mussels	Blocking skeletal muscle contraction	1	[65]

**Table 2:** Topical reviews on fumonisins over the last five years.

Subject	Content	Reference
Assessment	Biomarkers, metabolism, and biomonitoring of fumonisins in human biological fluids	[174]
Assessment	Impact on agriculture, food, and human health and their management strategies	[16]
Assessment	Risk assessment and intervention models for fumonisin of maize in South Africa	[175]
Assessment	Fumonisins and related *Fusarium* occurrence in wheat and its by-products	[176]
Assessment	Fumonisins and their modified forms	[177]
Assessment	Biological methods for fumonisins reduction and related *Fusarium* species control	[178]
Assessment	Fumonisins and *A. alternata* f. sp. *Lycopersici* (AAL) toxins in ruminants and their forages	[19]
Biosynthesis	Genetic regulation of fumonisins biosynthesis by specific genes and global regulators	[179]
Biosynthesis	Impact of environmental variables and genetics of maize resistance on fumonisin accumulation	[180]
Detection	Analytical methods for fumonisins detection in single corn kernels	[181]
Detection	Molecular methods for early detection of fumonisin-producing *F. verticillioides*	[182]
Plant resistant	Genomic, genes, and pathways in maize resistance to *Fusarium* ear rot and fumonisin accumulation	[183]
Plant resistant	Relationship between Bt maize hybrids and fumonisins contamination level	[184]
Toxicity	Mitochondrial toxicity induced by FB_1_	[185]
Toxicity	Molecular mechanisms underlying FB_1_-mediated toxicities and related interventions	[186]
Toxicity	CerS inhibition by fumonisins result in animal and plant disease	[187]
Toxicity	Dietary fumonisin and growth impairment in children and animals	[188]
Toxicity	Impact of fumonisin-contaminated feed on pig intestinal health	[189]
Toxicity	Oxidative stress-mediated toxicity and metabolism in vivo and in vitro	[190]

**Table 4:** Difference in genomic context of fumonisin biosynthetic gene (*fum*) cluster between strains of *Aspergillus* spp.

Fungi	Stains	*fum* Cluster	Reference
*Asp. niger*	fumonisin-producing strains	*fum* cluster	[208]
	fumonisin-non-producing strains	Intact *fum* cluster	[7,235]
*Asp. welwitschiae*	fumonisin-producing strains	*fum* cluster	[7]
	fumonisin-non-producing strains	Three *fum* cluster types including an intact cluster	[7,235]
*Asp. tubingensis*	fumonisin-producing strains	Not tested	[227]
	fumonisin-non-producing strains	Multiple patterns of *fum* gene deletion	[7]
*Asp. brasiliensis*	fumonisin-non-producing strains	Multiple patterns of *fum* gene deletion	[7]
*Asp. luchuensis*	fumonisin-non-producing strains	Multiple patterns of *fum* gene deletion	[7,236]
*Asp. fumigatus*	fumonisin-non-producing strains	Not detected	[208]
*Asp. oryzae*	fumonisin-non-producing strains	Not detected	[208]
*Asp. nidulans*	fumonisin-non-producing strains	Not detected	[208]
*Asp. foetidus*	fumonisin-producing strains	Not tested	[227]
